# Protocol of a prospective study on the diagnostic value of transcranial duplex scanning of the substantia nigra in patients with parkinsonian symptoms

**DOI:** 10.1186/1471-2377-7-28

**Published:** 2007-09-04

**Authors:** Annemarie MM Vlaar, Angela EP Bouwmans, Marinus JPG van Kroonenburgh, Werner H Mess, Selma C Tromp, Piet GWM Wuisman, Alfons GH Kessels, Ania Winogrodzka, Wim EJ Weber

**Affiliations:** 1 Department of Neurology, University Hospital Maastricht, P.O. Box 5800, 6202 AZ Maastricht, The Netherlands; 2 Department of Nuclear Medicine University Hospital Maastricht, P.O. Box 5800, 6202 AZ Maastricht, The Netherlands; 3 Department of Clinical Neurophysiology, University Hospital Maastricht, P.O. Box 5800, 6202 AZ Maastricht, The Netherlands; 4 Department of Clinical Neurophysiology, Maasland Hospital, P.O. Box 5500 6130 MB Sittard, The Netherlands; 5 Department of Clinical Epidemiology and Technology Assessment, University Hospital Maastricht, P.O. Box 5800, 6202 AZ Maastricht, The Netherlands

## Abstract

**Background:**

Parkinson's disease (PD) is the second most common neurodegenerative disorder. As there is no definitive diagnostic test, its diagnosis is based on clinical criteria. Recently transcranial duplex scanning (TCD) of the substantia nigra in the brainstem has been proposed as an instrument to diagnose PD. We and others have found that TCD scanning of substantia nigra duplex is a relatively accurate diagnostic instrument in patients with parkinsonian symptoms. However, all studies on TCD so far have involved well-defined, later-stage PD patients, which will obviously lead to an overestimate of the diagnostic accuracy of TCD.

We have therefore set out to conduct a prospective study testing the diagnostic accuracy of TCD in patients with a parkinsonism of unclear origin.

**Methods/Design:**

We will enrol 250 consecutive patients, who are referred to neurology outpatient clinics of two teaching hospitals, for analysis of clinically unclear parkinsonism. Patients, whose parkinsonism is clearly diagnosable at the first visit, will be excluded from the study. All patients will undergo a TCD of the substantia nigra. As a surrogate gold standard we will use the consensus clinical diagnosis reached by two independent, blinded, movement disorder specialist neurologists after 2 years follow-up. At the time of TCD, patients will also undergo a SPECT scan of the brain.

**Discussion:**

As this prospective trial enrols only patients with an early-stage parkinsonism, it will yield data on the diagnostic accuracy of TCD that is relevant to daily clinical practice: The neurologist needs a diagnostic tool that provides additional information in patients with a clinically indefinable parkinsonian syndrome. The above described observational longitudinal study was designed to explicitly study this aspect in the diagnostic process.

**Trial registration:**

(ITRSCC) NCT00368199

## Background

Parkinson's disease (PD) is the second most common neurodegenerative disorder with a life-time risk of 2 percent in males and 1.3 percent in females [1]. Diagnosis is based on clinical criteria. In most cases the diagnosis of PD is straightforward when cardinal clinical signs and symptoms as bradykinesia, rigidity, and resting tremor are present [2]. However, these main features of PD are shared, at least in part, by atypical parkinsonian syndromes (APS), like multi system atrophy (MSA), progressive supranuclear palsy (PSP) and corticobasal degeneration), essential tremor (ET), vascular parkinsonism (VP), drug induced parkinsonism (DIP), dementia with Lewy bodies and Alzheimer's disease. Besides delineating PD from the above parkinsonian syndromes, distinguishing PD from normality can also be difficult, especially in early stage of the disease [3].

The gold standard for the diagnosis of PD is post-mortem neuropathological examination. Clinicopathological studies show that 2–25 % of the patients with IPD are classified incorrectly in the final stage of their disease, even by specialists in movement disorders, with MSA and PSP accounting for most false positives [2,4,5]. Diagnostic accuracy is certainly less than 90% in earlier disease, as Litvan et al. found that the median sensitivity for the diagnosis of PD increased from 73% at the first visit to 80% to the last visit after a mean follow-up of 9 years, and the median positive predictive value increased from 46 to 64% [6].

A reliable test to diagnose PD is important for two reasons. Prognosis and medical treatment in the various parkinsonian syndromes differ considerably and an objective disease marker would facilitate the development of neuroprotective therapies [7,8]. Several procedures have been proposed to diagnose PD: functional imaging with Positron Emission Tomography (PET)-scan or Single Photon Emission Computer Tomography (SPECT), olfactory- and neuropsychological tests, and DNA tests [9-12].

At the moment neuro-imaging techniques like PET and SPECT are the most widely used diagnostic tools [8]. PET is at least as reliable as SPECT, but its use in routine clinical practice is limited by high costs and a relative short half-life of its radioactive tracers [13-16]. Despite its widespread use, there is no consensus about the value of SPECT scintigraphy in the differential diagnosis of PD [8,17-19]. The ability of SPECT scanning to discriminate PD from normality and other parkinsonian syndromes varies greatly among different studies. A major issue here is that many studies use well-defined later-stage patients that are obviously not representative for the diagnostic problem that one wants to solve with a SPECT.

A more recent addition to the diagnostic armamentarium of the neurologist is transcranial duplex scanning (TCD) of the substantia nigra (SN) in the brainstem. In 1994 Becker discovered that patients with PD had bilateral hyperechogenicity of the SN [12], probably caused by iron deposition [20,21]. Several publications confirmed this observation that up to 90% of PD patients have increased echo-intensity of the SN. In healthy subjects and in patients with ET or VP this hyperintensity of the SN is only found in 10–25% [20,22-29].

This technique has high inter-observer reliability [22,25,29]. In a pilot study with 45 patients with PD or APS who underwent SPECT and TCD we found a positive predictive value of 95% of an abnormal TCD for an abnormal FP-CIT SPECT scan [30].

Based on this study we hypothesized that TCD of substantia nigra is a tool deserving a place in the diagnostic work-up of PD/Parkinsonism patients. TCD is less costly and less invasive than SPECT [31]. Since a diagnostic test for parkinsonian syndromes is especially valuable in the early stage of disease(s), we devised a prospective diagnostic study with a clinical follow-up after 2 years as surrogate gold standard. As SPECT is currently the most widely used diagnostic tool in parkinsonian syndromes we included this in the study to directly compare the two techniques as to their diagnostic capacities in this field.

## Methods/Design

### Design

Observational, prospective, longitudinal study.

### Setting

Consecutive patients will be recruited from the Neurology Outpatient Clinic of two hospitals: the University Hospital Maastricht in Maastricht and the Maasland Hospital in Sittard, The Netherlands. TCD will be done in the departments of Clinical Neurophysiology of the two above mentioned hospitals. SPECT scanning will be done in the departments of Nuclear Medicine of the two hospitals

### Ethical approval

The Institutional Review Board (IRB) of the University Hospital Maastricht has approved the study (MEC 05–228, 4 April 2006). (This IRB also functions as IRB for the Maasland Hospital in Sittard, The Netherlands). All patients will be asked for informed consent through a standardised information form that is also approved by the Institutional Review Board.

### Participants

250 consecutive patients with new parkinsonian signs and symptoms (of unclear origin at the time of visit) referred to the Neurology Outpatient Clinics of the University Hospital Maastricht (n = 150) and the Maasland Hospital Sittard, (n = 100).

#### Inclusion criteria

1. Patients with parkinsonian signs and symptoms of unclear origin at the time of visit at the Neurology Outpatient Clinic. In his/her differential diagnosis the treating neurologist should be considering one of the following conditions: PD, MSA, and PSP. ET, VP or DIP.

2. Age older than 18 years.

#### Exclusion criteria

1. Patients presenting with a clear unequivocal diagnosis of their parkinsonism.

2. Patients whose life expectancy is less than the required follow-up of two years.

### Methods

After informed consent all subjects will undergo a structured interview, neurological examination (See Additional file 1), TCD and SPECT within 6 weeks of the initial visit at the Outpatient Clinic. After two years all patients are re-examined by two movement disorder specialist neurologists for a final clinical diagnosis. This diagnosis serves as a surrogate gold standard to calculate the accuracy of SPECT and TCD to differentiate between PD and other types of parkinsonism.

### A. Interview and neurological examination

After informed consent the patient is seen on the outpatient clinic for the inclusion interview and neurological examination by a third party physician (i.e. a physician not treating the patient, and blinded for information in the routine clinical records). In the structured interview a standard form with the following items are discussed: medical history, used drugs and effect, intoxications, duration of complaints, and most affected body- side (See attachment 1). The following clinimetric scales are scored: UPDRS (parts I, III and IV) [32], Hoehn and Yahr score [33], Hamilton Rating Scale [34] for depression and the SCOPA cognition scale [35]. The Sniffin Sticks smell test is done according to a standardised protocol [36,37]. Finally, the including physician will try to reach a probable diagnosis, strictly applying the UK Parkinson's Disease Society Brain Bank criteria [2].

### B. SPECT

All subjects will undergo SPECT scanning within 6 weeks of inclusion in the study. In this study FP-CIT (^123^I-ioflupane, Nycomed, Amersham, U.K.) is used as presynaptic radiotracer. Medication (amphetamine, citalopram, fentanyl, cocaine, fluoxetine, fluvoxamine, paroxetine, sertraline, venlafaxine) which could interfere with the radiotracer is discontinued at least 5 half life times. After intravenous injection of the tracer, SPECT measures baseline dopamine transporter integrity in the brain. SPECT is performed with a triple head camera (MultiSPECT3, Siemens, Ohio, USA) equipped with high-resolution collimators. A semi-automatic template model programme is used to calculate the ratios between left striatal and right striatal and occipital regions respectively. Total time of acquisition is 30 minutes (45 seconds per frame for 40 views per detector). Zoom factor: 1.00 and the matrix size: 128 × 128. Filtered back-projection acquisition is performed. Images are filtered using a Butterworth filter with a cut-off value of: 0.4–0.5 and an order of 5. A division between the caudate nucleus and putamen is made. The ratios are corrected using Alderson's brain phantom, with known activities in the caudate nucleus and putamen. A binding of two standard deviations below healthy controls is considered as abnormal (FP-CIT 8.25 +-1.85 for putamen and 7.76 +-1.77 for caudate nucleus). Beside quantitative the scans will be also judged visually by the same nuclear specialist blinded for the final clinical diagnosis. If quantitative and visual judgments do not agree the conclusion of visual judgment is taken (unpublished data).

### C. Transcranial Duplex Scanning (TCD)

TCD investigation is performed bilaterally through the pre-auricular bone window with a 2–4 MHz phased array transducer (SONOS 5500; Philips, Eindhoven, the Netherlands) by an experienced sonographer, blinded for the clinical data and SPECT results. The quality of the temporal bone window, the SN and Raphe nuclei (RN) of all subjects are scored directly by the sonographer blinded for the final clinical diagnosis and SPECT result. The quality of the bone window is scored as good, moderate or inconclusive.

Two different methods are applied for the evaluation of the echointensity of the SN. Firstly, the presence or absence of an obviously visible bilateral hyperechogenic SN is scored (qualitative method). The SN are scored as hyperechointens, not hyperechointens or inconclusive (= no typical configuration of hyperechointensity or low quality of the temporal bone window). Secondly, the area of an eventually hyperechogenic SN will be measured quantitatively (quantitative method). Both the right and left SN are measured from both sides, i.e. both temporal bone windows. After encircling, the area is automatically calculated. A hyperechogenic area of at least 0.2 cm^2 ^is classified as characteristic for PD. The RN are scored as: invisible (= iso-intense), just visible, visible (= hyper-intense) or inconclusive (= doubtful echointensity or low quality temporal bone window).

To determine inter-observer variability and to increase the power of the study a second sonographer will also judge the acquired echo data. A loop of 64 images of each patient will be acquired scanning the brainstem cranio-caudally and will be stored in order to allow for off-line analysis. Off-line the quality of the temporal bone window, SN and RN will scored by the second sonographer.

### D. Regular Outpatient follow-up

The initial treating neurologist will remain responsible for the regular outpatient management of the patient included in the study. He or she will discuss the test results with the patient, and base his/her treatment plan on these. All further clinical decisions will be made by the treating neurologist.

### E. Re-examination at two-year follow-up

Two years after inclusion, all patients will be re-examined separately by two independent movement disorder specialist neurologists blinded for the tests results. They will also be blinded for the clinical records of the treating neurologist. The same standard form as in the first visit is filled in (see Additional file 1) and they will be asked to reach a clinical diagnosis, independently from each other, according to generally accepted clinical criteria [38-44]. If these two diagnoses are not identical, the final diagnosis of this patient will be coded as inconclusive.

### Data analysis

Our main hypothesis is that TCD is as sensitive as SPECT to differentiate PD from other parkinsonian disorders. For the power analyses we assumed a sensitivity of SPECT of 90%, based on the analysis of our own data on 248 consecutive patients [19]. Assuming this 90% for TCD sensitivity we can accept as lowest border of the 95% confidence interval, 86% or higher:

*SD *= (√ (p1 (1-p1))/n → p1 = 0.9, sd 0.02, implying n = 190 patients, needed who have the hyperintensity of SN with TCD scanning.

In our pilot study 15% of the patients had an insufficient temporal bone window, so 224 patients are needed to compensate for the amount of inconclusive TCDs [28]. Based on this study we expect that 90% of all patients with inconclusive parkinsonism will ultimately have PD, so we set a target of initial 250 patients with unclear parkinsonism needed for this trial. We will calculate the sensitivity and specificity, positive predictive value, negative predictive value and diagnostic odd's ratio (OR) with its 95% confidence intervals (95% CI) of the first clinical judgment, TCD, FP-CIT SPECT scan and smell tests to predict the clinical diagnosis after 2 year follow-up. Accuracy is determined for all parkinsonian subgroups separately (PD versus APS, PD versus ET, and PD versus VP, PD versus DIP, and PD versus all other types of parkinsonism). For expected SPECT, TCD and smell tests scores for each parkinsonian disorder, see table 1.

**Table 1:** Expected TCE, FP-CIT SPECT and odour recognition results for all parkinsonian disorders

Disease	FP-CIT SPECT abnormal, [19, 46]	abnormal SN TCD [47]	odour recognition deficit	cognition deficit
IPD	++	+++	+++	±
ET	Normal	normal	normal	normal
VP	normal *	normal	normal (?)	±
DIP **	normal (+)	normal (+)	normal (+)	normal (±)
MSA	++	±	+	±
PSP	++	± (?)	normal (±)	++

= normal FP-CIT tracer binding ratios

↓: FP-CIT binding of at least 2 sd. below healthy controls

* especially visual judgement together with CT or MRI

** At least 10% percent of the patients with DIP will develop to the PD [48]. So some patients in early stages of PD can theoretically present as DIP. Berg et al. investigated the relation between echointensity of SN on TCD and DIP after the start of antipsychotic drugs. Patients with serious parkinsonism scored higher echointensity as patients with mild or no parkinsonism [47].

*** In our retrospective trial 76% of the 27 patients with APS had FP-CIT binding lower as 2 standard deviations below healthy controls. In the 11 studies included in our meta-analysis this percentage varies form 67 to 100% [19, 46]

Additionally we will determine the predictive value of TCD compatible with PD for an abnormal FP-CIT SPECT scan. Finally the inter-observer reliability for SN and RN judgement by TCD will be determined (Cohen's kappa test).

SPSS 11.0 for windows (SPSS, Chicago, IL, USA) and StataSE9 (Stata corporation, Texas, USA) will be used for statistical analysis.

## Discussion

The hitherto published literature on TCD in parkinsonian syndromes are cross-sectional studies on clinically well-defined patient populations [20-30,45]. Although one has to start with these to study the diagnostic potential of a new technique, these kind of studies are obviously not representative of the clinical problem that one wants to solve with a TCD in parkinsonian syndromes. The treating neurologist wants a diagnostic tool that provides additional information in patients with a clinically indefinable parkinsonian syndrome.

The above described observational longitudinal study was designed to explicitly study this aspect in the diagnostic process. We arbitrarily choose the clinical diagnosis after two years as the surrogate gold standard. This is, of course, not ideal, as there will always remain a small proportion of patients that is not definitely diagnosable after two years, and misdiagnoses (as opposed to the ultimate gold standard the post-mortem pathological analysis) are still possible. In an effort to tackle his last obstacle we require the final diagnosis to be shared by two independent, blinded, experienced movement disorders specialist neurologists.

We included SPECT scans in the study to enable us to make a direct comparison between SPECT and TCD as to their diagnostic accuracy. Although the use of SPECT scans in the diagnostic work-up of parkinsonian patients is still debated, it is widely used [8,19,46]. We feel that this will add to the clinical relevancy of our study results. Additionally, contributions of tests for smell, depression, cognition, in the diagnostic process can be assessed also.

### Duration and expected study completion

4 years (2 years inclusion, 2 follow-up). Start recruiting 1-9-2006. Expected study completion date 1-10-2010.

## Abbreviations

PD, Parkinson's disease; MSA, multiple system atrophy; PSP, progressive supranuclear palsy; VP, vascular parkinsonism; DIP, drug induced parkinsonism; ET, essential tremor; SPECT, Single Photon Emission Computer Tomography; PET, Positron Emission Tomography; FP-CIT, ^123^I-ioflupane; TCD, transcranial duplex scanning; SN, substantia nigra; RN, Raphe nuclei; UPDRS, United Parkinson's Disease Rating Scale

## Competing interests

The author(s) declare that they have no competing interests.

## Authors' contributions

All authors have read and approved the final manuscript. WM and WW initiated the study. AV, WW, ST, AK, and WW wrote the protocol. AV, AB, AW and WW will do the patient inclusion. AW will do the clinical examination after two years for a final diagnosis. AV, WW and AK will do the statistical calculations. AV, AB, AW, WW, WM, MK and ST will write the first draft of the paper.

## Pre-publication history

The pre-publication history for this paper can be accessed here:



## Supplementary Material

Additional file 1 Standard clinical scorings form. Standard form used to collect clinical data as described. Click here for file
