# Supplementary material for: Protocol of a prospective study on the diagnostic value of transcranial duplex scanning of the substantia nigra in patients with parkinsonian symptoms
Source: BMC Neurol. 2007 Sep 4;7:28. doi: 10.1186/1471-2377-7-28 (PMC2034584; doi:10.1186/1471-2377-7-28)
Supplement: Additional file 1 — Standard clinical scorings form. Standard form used to collect clinical data as described. [file 1471-2377-7-28-S1.doc]

***Additional file***

***Standard clinical scorings form***

**general:**

Age:

Referrer:

Investigator:

Date of investigation:

**Anamnesis:**

1. Medical history:

2. Medication:

Anti-parkinsonian drugs in present and past:

Effect of anti-parkinsonian drugs (positive, negative, unclear, inadequate dose)? Adequate dose of anti-parkinsonian drugs (at least 1000mg l-dopa daily):

3. Intoxications (alcohol, smoking, drugs):

4. Durations of complaints:

5. Most affected body-side:

**CLINIMETRIC SCALES:**

# uk BRAIN BANK criteria [2]:

# United Parkinson’s Disease Rating Scale (part I, III, IV) [30]:

# hoehn and YAHR score [31]:

# SCOPA cognition scale [33]:

# Hamilton rating scale for depression [32]:

# Sniffin sticks smell test [34] [35]:

#

**Most probableclinical diagnosis:**

1. Idiopathic Parkinson’s disease:

2. Essential tremor

3. Vascular parkinsonism

4. Drug-induced parkinsonism

5. Multiple system atrophy

6. Progressive supranuclear palsy

7. Diffuse Lewy body disease

8. Corticobasal degeneration

9. Other:

**DEGREE OF CONFIDENCE:**

Certain / Not certain
